# Progress in the application of cardiac magnetic resonance to predict recurrence of atrial fibrillation after catheter ablation: A systematic review and *meta*-analysis

**DOI:** 10.1016/j.ijcha.2025.101603

**Published:** 2025-01-17

**Authors:** Cuncun Yu, zhenjuan Liu, shiyu Zhu

**Affiliations:** a Department of Emergency, Qilu Hospital (Qingdao), Cheeloo College of Medicine, Shandong University, Qingdao 266000 People's Republic of China; b Department of Critical Care Medicine, Qingdao Hospital, University of Health and Rehabilitation Sciences (Qingdao Municipal Hospital), Qingdao 266000 People's Republic of China; c China Medical University, Shenyang 110000 People's Republic of China

**Keywords:** Atrial fibrillation, Cardiac magnetic resonance, Recurrence, catheter ablation

## Abstract

**Background:**

This systematic review and *meta*-analysis aimed to assess changes in left atrial structure and function at baseline and after catheter ablation and their association with atrial fibrillation recurrence using cardiac magnetic resonance imaging (MRI).

**Methods:**

As of June 2024, a total of 3086 articles have been obtained through searching PubMed, Embase, and Cochrane databases. Standard mean differences and 95% confidence intervals were used to examine structural and functional changes of left atrium after catheter ablation and their relationship with recurrence of atrial fibrillation.

**Results:**

A total of 13 prospective cohort studies were included in the analysis. Decreased left atrial emptying capacity is seen in the short term after catheter ablation, and structural changes in the left atrium are seen in the long term (EFActive: SMD, 1.23, 95 % CI, 1.10–2.36, p < 0.05; EFTotal: SMD, 0.83, 95 % CI, 0.02–1.64, p < 0.05; MinLAV: SMD, 0.30, 95 % CI, 0.01–0.59, p < 0.05). Decrease in left atrial volume after catheter ablationis positively associated with the risk of recurrence of atrial fibrillation. (MaxLAV: SMD, 1.27, 95 % CI, 0.05, 2.49, p < 0.05; MaxLAVi: SMD, 0.48, 95 % CI, 0.05,0.9, p < 0.05;MinLAVi: SMD, 0.78, 95 % CI, 0.39,1.16, p < 0.05). The larger the left atrial volume and the lower the emptying and strain function, the greater the likelihood of recurrence of atrial fibrillation following catheter ablation, (MaxLAV: SMD, 0.38, 95 % CI, 0.18,0.59, p < 0.05;MinLAV: SMD, 0.83,95 % CI, 0.41,1.24, p < 0.05; MaxLAVi: SMD, 0.35, 95 % CI, 0.21,0.50, p < 0.05;MinLAVi: SMD, 0.62, 95 % CI, 0.47,0.78, p < 0.05; EFPassive: SMD, −0.57, 95 % CI, −0.78, −0.37, p < 0.05; EFActive: SMD, −0.62, 95 % CI, −1.08, −0.15, P < 0.05; EFTotal: SMD, −0.70, 95 % CI, −0.97, −0.44, P < 0.05; ℇCT: SMD, −0.61, 95 % CI, −0.90, −0.32, p < 0.05; PLAS: SMD, −1.22, 95 % CI, −1.87, −0.57, p < 0.05; ℇR: SMD, −0.50, 95 % CI, −0.79, −0.21, p < 0.05; PLAS: SMD, −1.22, 95 % CI, −1.87, −0.57, p < 0.05).

**Conclusion:**

Short-term left atrial functional impairment can be observed after catheter ablation, while long-term reduction in left atrial volume can be seen. Changes in left atrial volume are likely to lead to the recurrence of atrial fibrillation, while alterations in left atrial function help maintain sinus rhythm. Larger left atrial volume and lower emptying and strain function at baseline assessment by cardiac magnetic resonance are more likely to lead to recurrence of atrial fibrillation after catheter ablation, which may be useful to identify those for whom catheter ablation has reduced success or for whom more aggressive ablation or medications may be useful.

## 1 Introduction

Atrial fibrillation refers to the disorder of atrial electrical activity caused by the loss of normal rhythm in the atrium, usually manifested as an irregular and fast heart rate. It leads to an increased risk of stroke and heart failure and is associated with a decline in quality of life. It has become a health burden in society today. Catheter ablation is recommended for patients with symptomatic, drug-refractory atrial fibrillation, because it can improve quality of life and reduce the burden of atrial fibrillation compared with medical treatment, and has greater safety and benefits [1], On the one hand, catheter ablation helps maintain sinus rhythm, which has been shown to improve left ventricular function. On the other hand, it creates new scars, which are related to poor LA function [2], [3], [4], [5]. Research on the impact of catheter ablation on LA size and function is inconclusive. Compared to cardiac ultrasound, cardiac magnetic resonance (CMR), with its higher spatial resolution, is considered the gold standard for assessing myocardial motion [6]. Despite advanced ablation catheters and technologies, the recurrence rate of atrial fibrillation after catheter ablation is still high. Risk stratification for catheter ablation in atrial fibrillation patients is complex and challenging, and there is still a lack of optimal patient selection methods. The purposes of this study were to 1) assess changes in LA structure and function during short- and long-term follow-up after catheter ablation; and 2) use cardiac magnetic resonance to detect potential predictors of atrial fibrillation recurrence.

## 2 Methods

Meta-analyses were conducted according to the Preferred Reporting Items for Systematic Reviews and Meta-analyses guidelines (PRISMA) [7].

### 2.1 Search strategy

We searched the PubMed, Embase, and Cochrane Library electronic databases for relevant studies until June 2024. The search terms used were as follows: (“Catheter Ablation” OR “Ablation, Catheter” OR “Ablation, Transvenous Electric”) AND (“Atrial Fibrillation” OR “Atrial Fibrillations” OR “Persistent Atrial Fibrillation” OR “Familial Atrial Fibrillation” OR “Paroxysmal Atrial Fibrillation”) AND(“Magnetic Resonance Imaging” OR “Imaging, Magnetic Resonance” OR “NMR Imaging”). Related details are provided in Supplementary S1.

### 2.2 Selection criteria

Studies were considered eligible if they met the following criteria: (1) prospective cohort studies; (2) patients diagnosed with atrial fibrillation; (3) all patients underwent catheter ablation and cardiac magnetic resonance imaging both before and after the procedure; (4) written in English. The exclusion criteria were: (1) *meta*-analysis, case report, and duplicates; (2) outcomes could not be extracted; and (3) inappropriate selection of study groups. Two researchers independently performed the screening and data extraction, and any disagreements were resolved by a third researcher.

### 2.3 Type of outcome

The outcome metrics of this *meta*-analysis were changes in left atrial structure and function following catheter ablation and its association with recurrence of AF. Additionally, the relationship between baseline left atrial structure and function and the recurrence of AF was assessed.

### 2.4 Data extraction

Two investigators (CY and SZ) independently conducted the literature search and data extraction. They extracted the following information: first author, publication year, sample size, follow-up time, left atrial structure sizes (MaxLAV, MinLAV, MaxLAVi, and MinLAVi) left atrial function (EFPassive, EFActive, EFTotal, ℇCT, ℇCD, ℇR and PLAS) at baseline and after catheter ablation, number of recurrences of atrial fibrillation.

### 2.5 Quality assessment of the literature

The quality of the included studies was based on the NewcastleOttawa scale for cohort studies. Each item, was evaluated in the following categories: selection (four items, 1 point each), comparability (one item, up to 2 points), and outcome (three items, 1 point each). Studies with a 5-points rating or higher were considered high-quality. Disagreements were resolved through discussion after two researchers (CY and SZ) independently reviewed eligibility.

### 2.6 Data analysis

We analyzed the data using STATA ES-64 Statistical significance was defined as p < 0.05 in the Z test. Continuous data are presented as mean ± standard deviation. forest plots for hazard ratios from relevant studies were constructed using the metan function. The choice of Pooling Model was based on the magnitude of heterogeneity. Heterogeneity was assessed using Cochrane’s Q-test and the I^2^ statistics. Cochran’s Q-test was used to evaluate between-study heterogeneity, while the I2 statistic quantified the degree of heterogeneity. Publication bias was assessed with funnel plots and the Egger test.

## 3 Results

### 3.1 Literature research

**Fig. 1:**
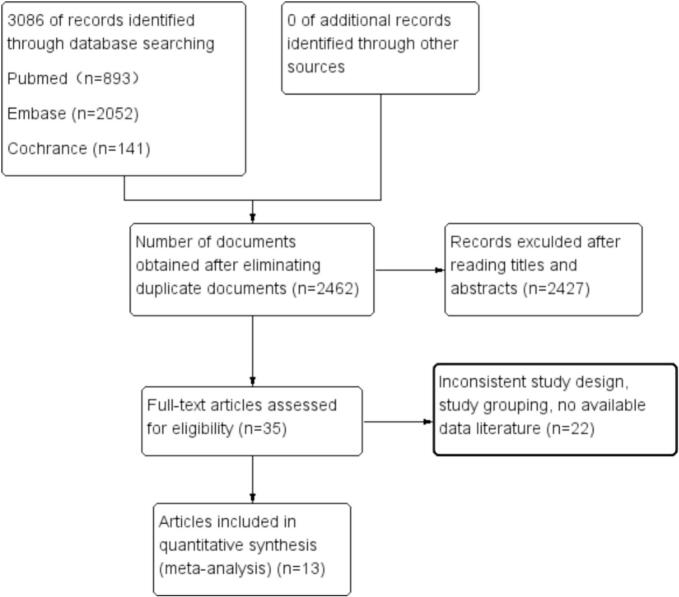
Flowchart of study selection.

A total of 3086 related literature were obtained through the preliminary search; After screening titles and abstracts, 624 duplicates and 2,427 irrelevant studies were excluded. Subsequently, 22 articles were removed due to issues with study design, grouping, or lack of available results. Finally, 13 studies were included in the analysis. A flow chart of the literature screening process is shown in Fig. 1.

### 3.2 Changes in left atrial structure and function after catheter ablation

**Fig. 2:**
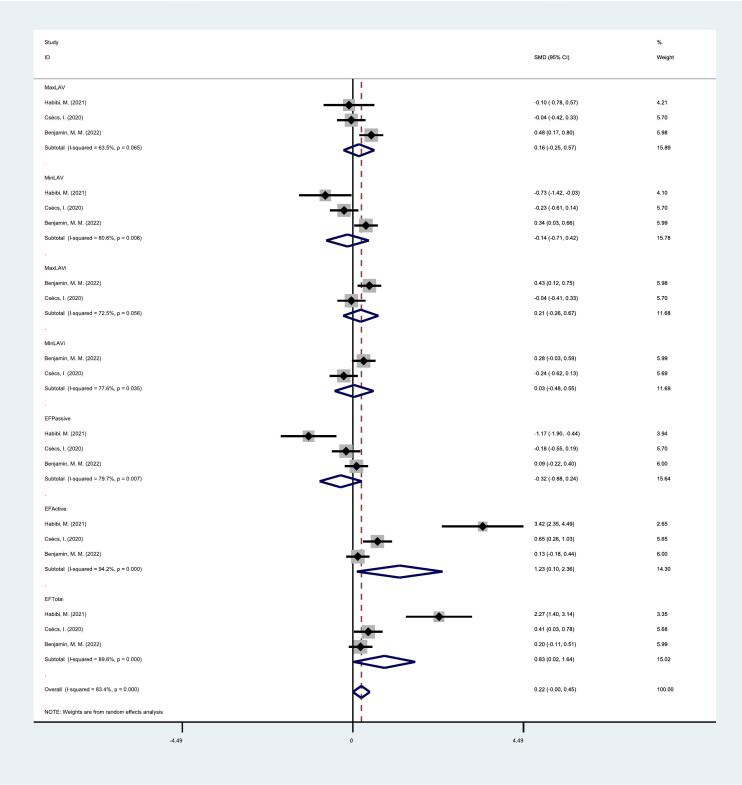
Forest plot of short-term changes in left atrial structure and function after catheter ablation.

**Fig. 3:**
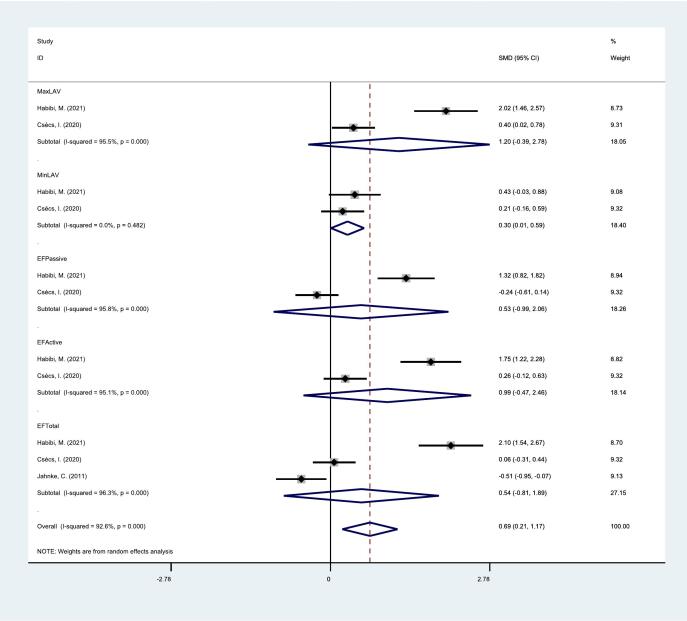
Forest plot of long-term changes in left atrial structure and function after catheter ablation.

This *meta*-analysis contained 4 studies that examined the short- and long-term associations between atrial fibrillation catheter ablation and left atrial structure and function. The results show that there is a decrease in left atrial emptying function in the short-term follow-up after catheter ablation (EFActive: SMD, 1.23, 95 % CI, 1.10–2.36, p < 0.05; EFTotal: SMD, 0.83, 95 % CI, 0.02–1.64, p < 0.05)(Fig. 2); There is an increase in left atrial minimum volume during long-term follow-up after catheter ablation (MinLAV: SMD, 0.30, 95 % CI, 0.01–0.59, p < 0.05) (Fig. 3), as shown in Table 1. Relevant sensitivity analyses and publication bias tests were not performed due to the limitations in the amount of literature.

**Table 1:** Structural and functional changes in the left atrium after catheter ablation.

	**Index**	**Number of** **studies**	**Heterogeneity test**		**SMD (95 %CI)**	**P −value**
			**P value**	**I^2^ value (%)**		
**Short Term Follow Up**	**MaxLAV**	**3**	**0.065**	**63.5**	**0.16** (**−0.25, 0.57**)	**0.444**
	**MinLAV**	**3**	**0.006**	**80.6**	**−0.14** (**−0.71, 0.42**)	**0.620**
	**MaxLAVi**	**2**	**0.056**	**72.5**	**0.21** (**−0.26, 0.67**)	**0.380**
	**MinLAVi**	**2**	**0.035**	**77.6**	**0.03** (**−0.48, 0.55**)	**0.907**
	**EFPassive**	**3**	**0.007**	**79.7**	**−0.32** (**−0.88, 0.24**)	**0.259**
	**EFActive**	**3**	**0.000**	**94.2**	**1.23** (**1.10, 2.36**)	**0.032**
	**EFTotal**	**3**	**0.000**	**89.6**	**0.83** (**0.02, 1.64**)	**0.045**
**Long Term Follow Up**	**MaxLAV**	**2**	**0.000**	**95.5**	**1.20** (**−0.39, 2.78**)	**0.139**
	**MinLAV**	**2**	**0.482**	**0**	**0.30** (**0.01,0.59**)	**0.043**
	**EFPassive**	**2**	**0.000**	**95.8**	**0.53** (**−0.99, 2.06**)	**0.492**
	**EFActive**	**2**	**0.000**	**95.1**	**0.99** (**−0.47, 2.46**)	**0.183**
	**EFTotal**	**2**	**0.000**	**96.3**	**0.54** (**−0.81, 1.89**)	**0.434**

MaxLAV: maximum volume; MinLAV: minimum volume; MaxLAVi: maximum volume index; MinLAVi: minimum volume index; EFPassive: passive ejection fraction; EFactive: active ejection fraction; EFTotal: Total ejection fraction

### 3.3 Relationship between baseline left atrial structure and function and atrial fibrillation recurrence after catheter ablation

Meta-analysis of the combined data from all the studies showed that there was no significant difference in the number of patients with paroxysmal AF in the recurrent AF group after catheter ablation compared with the non-recurrent AF group, which suggests that the different subgroups of AF have no effect on the recurrence of AF after catheter ablation(RR**,** 0.91**,** 95 %CI (0.80,1.34), P > 0.05). Related details are provided in Supplementary S1.

This *meta*-analysis contained 13 studies that examined the relationship between baseline left atrial structure and function and atrial fibrillation recurrence after catheter ablation. as shown in Table 2. Various indicators related to left atrial structure include MaxLAV, MinLAV, MaxLAVi, and MinLAVi. The larger it is at baseline, the more likely it is to cause recurrence of atrial fibrillation after catheter ablation. (MaxLAV: SMD, 0.38, 95 % CI, 0.18,0.59, p < 0.05;MinLAV: SMD, 0.83,95 % CI, 0.41,1.24, p < 0.05; MaxLAVi: SMD, 0.35, 95 % CI, 0.21,0.50, p < 0.05;MinLAVi: SMD, 0.62, 95 % CI, 0.47,0.78, p < 0.05), Various indicators related to left atrial function include EFPassive, EFActive, and EFTotal. However, recent relevant literature has regarded left atrial strain assessment as a new measurement of left atrial function. Various traditional indicators representing left atrial function include EFPassive, EFActive, and EFTotal. Recent studies have used left atrial strain assessment as a new measurement method of left atrial function, including ℇR, ℇCT, ℇCD, and PLAS. The research results show that lower left atrial evacuation and strain functions at baseline promotes recurrence of atrial fibrillation after catheter ablation. (EFPassive: SMD, −0.57, 95 % CI, −0.78, −0.37, p < 0.05; EFActive: SMD, −0.62,95 % CI, −1.08, −0.15, P < 0.05; EFTotal: SMD, −0.70, 95 % CI, −0.97, −0.44, P < 0.05; ℇCT: SMD, −0.61, 95 % CI, −0.90, −0.32, p < 0.05; PLAS: SMD, −1.22, 95 % CI, −1.87, −0.57, p < 0.05; ℇR: SMD, −0.50, 95 % CI, −0.79, −0.44, p < 0.05; ℇCD: SMD, −0.20, 95 % CI, −0.49,0.08, p > 0.05; PLAS: SMD, −1.22, 95 % CI, −1.87, −0.57, p < 0.05).

**Table 2:** Characteristics of studies included.

**Author**	**Year**	**Methods**	**Number of atrial fibrillation**	**prognosis**	**Follow-Up**	**Detection Methods**	**Pre-ablation**	**Post-ablation**
Benjamin, M. M. [8]	**2022**	**PVI**	**AF** (**n = 80**)	**Recurrence(n = 21):** **No recurrence(n = 59)**	**4 years**	**12-lead ECG, event recording, or Holter monitor**	**R > NR** **MaxLAV、MinLAV、MinLAVi、MaxLAVi、EFPassive** **R < NR** **EFTotal、EFActive、ℇR、ℇCT、ℇCD**	**R > NR** **MaxLAV、MinLAV、MinLAVi、MaxLAVi、ℇCD** **R < NR** **EFActive EFPassive 、EFTotal 、ℇR、ℇCT、**
Hopman, Lhga [9]	**2023**	**PVI**	**AF** (**n = 110**)	**Recurrence(n = 39):** **No recurrence(n = 71)**	**1,3,6,12 months**	**additional ECG recordings or 24–48 h** **Holter-monitoring**	**R > NR** **MinLAV 、MaxLAV 、MinLAVi、MaxLAVi** **R < NR** **EFTotal 、ℇR、ℇCT、ℇCD**	
Habibi, M. [10]	**2021**	**catheter ablation**	**AF** (**n = 51**)	**Recurrence(n = 16):** **No recurrence(n = 22)**	**,3,6,12 months**	**Symptoms patients** **a 24-hour ambulatory** **ECG monitor or 30-day event monitor** **No symptoms patients** **ECG and/or up to 7-day monitoring**	**R > NR** **MaxLAV、MinLAV** **R < NR** **EFActive 、EFPassive 、EFTotal 、PLAS**	**R > NR** **MaxLAV、MinLAV** **R < NR** **EFActive 、EFPassive 、EFTotal 、PLAS**
Csécs, I. [11]	**2020**	**catheter ablation**	**AF** (**n = 55**)	**Recurrence(n = 24):** **No recurrence(n = 26)**	**1,2,3,6,9,12 months**	**1,2,9months** **telephone interviews(12-lead ECG** **and 7-day Holter monitors)** **3,6,12monrhs** **12-lead ECG and 7-day Holter monitors**	**R > NR** **MaxLAVi、MinLAVi、** **R < NR** **EFTotal、EFActive、EFPassive、PLAS**	**R > NR** **MaxLAVi、MinLAVi 、EFTotal、EFPassive、EFActive、PLAS**
Dodson, J. A. [12]	**2014**	**PVI**	**AF** (**n = 346**)	**Recurrence(n = 124):** **No recurrence(n = 222)**	**3- to 6-month intervals**	**Symptoms patients** **cardiac monitoring** **No symptoms patients** **electrocardiography**	**R < NR** **EFpassive、EFtotal**	
Habibi, M. [13]	**2016**	**radiofrequency ablation**	**AF** (**n = 121**)	**Recurrence(n = 52):** **No recurrence(n = 69)**	**every 3 months**	**Symptoms patients** **24-hour holler monitoring or 30-** **day event monitoring** **No symptoms patients(3,6,12 months)** **ECG and/or 1**–**7 day monitoring**	**R > NR** **MinLAV 、MaxLAV 、MinLAVi、MaxLAVi、EFActive** **R < NR** **EFTotal、EFPassive、PLAS**	
Nakamori, S. [14]	**2018**	**PVI**	**AF(n = 227)**	**Recurrence(n = 88):** **No recurrence(n = 139)**	**3- to 6-month**	**All patients** **resting electrocardiography** **symptoms patients** **cardiac monitoring**	**R > NR** **MaxLAV、MinLAV、MinLAVi、MaxLAVi** **R < NR** **EFTotal、EFActive、EFPassive**	**R > NR** **MaxLAV、MaxLAVi**
M Gastl [15]	**2022**	**PVI**	**AF(n = 52)**	**Recurrence(n = 12):** **No recurrence(n = 40)**	**3,6,12 month**	**24–72 h Holter monitoror12-lead electrocardiogram**	**R < NR** **MaxLAV、EFTotal、EFActive、EFPassive、ℇR、ℇCT** **R > NR** **ℇCD**	
Ciuffo, L. [16]	**2019**	**catheter ablation**	**AF** (**n = 208**)	**Recurrence(n = 101):** **No recurrence(n = 107)**	**6 months**	**24-hour Holter monitor or a 30-day event monitor**	**R > NR** **minLAV、maxLAV** **R < NR** **EFTotal、EFPassive、EFActive**	
Hof, I. E. [17]	**2014**	**PVI**	**AF** (**n = 206**)	**Recurrence(n = 37):** **No recurrence(n = 169)**	**1,3,6,12,24 months**	**1,3,6,12,24 months** **ECG** **3 and 6 months** **48-h Holter monitoring w**	**R > NR** **maxLAV**	
Chubb, H. [18]	**2019**	**catheter ablation**	**AF(n = 89)**	**Recurrence(n = 31):** **No recurrence(n = 58)**	**6,12 months and yearly thereafter**	**No symptoms patients** **12-lead ECG ± Holter monitor** **Symptoms patients** **12-lead ECG, Holter monitor or event** **monitor assessment**	**R > NR** **maxLAV** **R < NR** **EFTotal**	
Gucuk Ipek, E. [19]	**2016**	**catheter ablation**	**AF** (**n = 216**)	**Recurrence(n = 22):** **No recurrence(n = 97)**	**3,6,12 months**	**24-hour Holter monitoring and scheduled electrocardiography**	**R > NR** **maxLAV、MaxLAVi** **R < NR** **EFTotal、EFPassive、EFActive、PLAS**	
Jahnke, C. [20]	**2011**	**PVI**	**AF** (**n = 41**)	**Recurrence(n = 10):** **No recurrence(n = 31)**	**1,3,6,12 months**	**periodic electrocardiograms at rest and repetitive 24 h Holter monitoring**	**R > NR** **EFTotal**	**R > NR** **EFTotal**

A sensitivity analysis was performed to assess the impact of each study on total SMD. There was no significant change in cumulative SMD after sequential removal of each individual study, indicating that the findings of this analysis were stable.

We used funnel plots and Egger's test to determine publication bias, and the result indicated that there was no clear publication bias (MaxLAV: 95 % CI, −4.44 to 5.93, p = 0.744;MinLAV: 95 % CI, −5.47 to 14.47p = 0.246; MaxLAVi: SMD95 % CI, −6.63 to 2.84, p = 0.351;MinLAVi: 95 % CI, −4.78 to 2.39, p = 0.408; EFPassive: 95 % CI, −3.13 to 3.96, p = 0.790; EFActive: 95 % CI, −10.89 to 2.18, p = 0.154; EFTotal: 95 % CI, −5.16 to 2.34, p = 0.421 ℇR: 95 % CI, −10.53 to 14.30p = 0.304; ℇCT: 95 % CI, –22.31 to 19.29, p = 0.526; ℇCD: 95 % CI, −30.52 to 33.27, p = 0.681;PLAS: 95 % CI, −18.99 to 7.39, p = 0.199).

PVI: Pulmonary vein isolation; AF: Atrial Fibrillation; R: recurrence; NR: no recurrence; MaxLAV: maximum volume; MinLAV: minimum volume; MaxLAVi: maximum volume index; MinLAVi: minimum volume index; EFPassive: passive ejection fraction; EFactive: active ejection fraction; EFTotal: Total ejection fraction; ℇCD, conduit strain; ℇCT, contractile strain; ℇR, reservoir strain; PLAS: peak longitudinal atrial strain

### 3.4 Relationship between left atrial structure and function after catheter ablation and atrial fibrillation recurrence

This *meta*-analysis contained 5 studies that examined the relationship between left atrial structure and function after catheter ablation and atrial fibrillation recurrence. as shown in Table 3. Larger left atrial volume can lead to recurrence of atrial fibrillation after catheter ablation (MaxLAV: SMD, 1.27, 95 % CI, 0.05,2.49, p < 0.05;MinLAV: SMD, 2.13,95 % CI, −1.01,5.28, p > 0.05; MaxLAVi: SMD, 0.48, 95 % CI, 0.05,0.9, p < 0.05;MinLAVi: SMD, 0.78, 95 % CI, 0.39,1.16, p < 0.05), Among various traditional indicators representing left atrial function, lower EFActive was associated with atrial fibrillation recurrence (EFActive SMD, −1.61, 95 % CI, **−**3.08, −0.13, p < 0.05), while EFTotal and EFPassive were not significantly correlated with atrial fibrillation recurrence(EFPassive: SMD, −0.71, 95 % CI, −1.50,0.09, p > 0.05; EFTotal: SMD, −1.38,95 % CI, −3.42,0.66, P > 0.05). Among all indicators of left atrial strain, lower PLAS after catheter ablation promotes recurrence of atrial fibrillation(PLAS: SMD, −2.08, 95 % CI, −3.71, −0.46, p < 0.05),

**Table 3:** Relationship between structural and functional changes in the left atrium before and after catheter ablation and recurrence of atrial fibrillation.

	**Index**	**Number of** **studies**	**Heterogeneity test**		**SMD (95 %CI)**	**P −value**
			**P value**	**I^2^ value (%)**		
Pre-ablation	MaxLAV	9	0.055	47.5	0.38 (0.18,0.59)	0.000
	MinLAV	5	0.002	75.9	0.83(0.41,1.24)	0.000
	MaxLAVi	7	0.284	19.1	0.35(0.21,0.50)	0.000
	MinLAVi	6	0.766	0	0.62(0.47,0.78)	0.000
	EFPassive	9	0.018	56.8	−0.57(−0.78, −0.37)	0.000
	EFActive	8	0.000	88.1	−0.62(−1.08, −0.15)	0.009
	EFTotal	12	0.000	78.2	−0.70(−0.97, −0.44)	0.000
	ℇR	3	0.818	0	−0.50(−0.79, −0.21)	0.001
	ℇCT	3	0.785	0	−0.61(−0.90, −0.32)	0.000
	ℇCD	3	0.680	0	−0.20(−0.49,0.08)	0.165
	PLAS	4	0.001	82.6	−1.22(−1.87, −0.57)	0.000
Post-ablation	MaxLAV	3	0.000	93.9	1.27(0.05,2.49)	0.042
	MinLAV	2	0.000	96.4	2.13(−1.01,5.28)	0.184
	MaxLAVi	3	0.061	64.2	0.48(0.05,0.9)	0.027
	MinLAVi	2	0.203	38.4	0.78(0.39,1.16)	0.000
	EFPassive	3	0.005	81.4	−0.71(−1.50,0.09)	0.08
	EFActive	3	0.000	93.1	−1.61(−3.08, −0.13)	0.033
	EFTotal	3	0.000	95.5	−1.38(−3.42,0.66)	0.184
	PLAS	2	0.004	88.2	−2.08(−3.71, −0.46)	0.012

A sensitivity analysis was performed to assess the impact of each study on total SMD. There was no significant change in cumulative SMD after sequential removal of each individual study, indicating that the findings of this analysis were stable.

We used funnel plots and Egger's test to determine publication bias, and the result indicated that there was no clear publication bias except for EFActive (MaxLAV: 95 % CI, −63.35 to 73.52, p = 0.518; MaxLAVi: 95 % CI, −51.76 to 49.19, p = 0.801; MinLAVi: 95% CI, −147.20 to 128.83, p =0.553; EFPassive: 95 % CI, −57.68 to 32.74, p = 0.177;EFActive: 95 % CI, −15.45 to −6.85, p = 0.019; EFTotal: 95 % CI, −122.73 to 105.45, p = 0.512;). The application of trim and fill method for analysis shows that the significance of the effect size has not changed significantly, and publication bias has little impact on the results of the meta-analysis.

## 4 Discussion

1. Short-term left atrial functional impairment can be observed after catheter ablation, and long-term reduction in left atrial volume can be seen.
2. Changes in left atrial volume are likely to lead to the recurrence of atrial fibrillation, while alterations in left atrial function help maintain sinus rhythm.
3. Larger left atrial volume and lower emptying and strain function at baseline assessment by cardiac magnetic resonance are more likely to lead to recurrence of atrial fibrillation after catheter ablation, which may be useful to identify those for whom catheter ablation has reduced success or for whom more aggressive ablation or medications may be useful.

### 4.1 Acute functional impairment and long-term structural changes

Catheter ablation is effective in reducing the recurrence of atrial fibrillation (AF), with an overall reduction of 48 %. However, the recurrence rate of symptomatic AF remains high at 51 % at 5 years [21], Early recurrence is thought to result from incomplete fibrous tissue formation around the pulmonary veins (PVI), allowing AF electrical impulses to leak. In contrast, late recurrence is often due to the shifting of AF foci from the pulmonary veins to the left atrium (LA), driven by stressors and comorbid conditions.

Recent studies have employed multiparametric and longitudinal evaluations of the left atrium's structure and function to predict AF prognosis and guide therapeutic decisions. The *meta*-analysis in this study found a positive correlation between increased left atrial volume and AF recurrence after ablation. Specifically, each 10 mL increase in left atrial volume was associated with a 1.07-fold increase in the risk of AF recurrence, with a hazard ratio of 1.07 (95 % CI: 1.03–1.12; I2 = 41.4 %). Structural features in this study included MaxLAV, MinLAV, MaxLAVi, and MinLAVi. Left atrial dilatation, a key marker of atrial remodeling, was identified as a prognostic factor for AF recurrence after ablation [22]. Notably, a decrease in minimum left atrial volume during long-term follow-up was observed, likely reflecting reverse remodeling after the procedure. In terms of functional characteristics, EFPassive, EFActive, and EFTotal were evaluated. Reduced left atrial emptying function is a contributing factor to AF, as it is associated with increased left atrial pressure and pulmonary vein distension, which in turn can lead to left atrial electrical remodeling [23]**.** Several studies have demonstrated that catheter ablation causes acute functional impairment of the left atrium(9, 11, 13, 14, 22, 24, 25). Emerging data also suggest a link between left atrial dysfunction and an increased risk of cardioembolic stroke. These findings underscore the importance of appropriate anticoagulation during the perioperative period to prevent such complications. Short-term follow-up after catheter ablation revealed left atrial wall edema and thickening, which were related to acute injury in EFActive and EFTotal. On the other hand, EFPassive was more closely associated with sclerosis of the left atrial wall due to increased fibrosis, which explains the lack of significant injury to EFPassive in the short-term follow-up.

### 4.2 Association of baseline left atrial structure and function with recurrence of atrial fibrillation after catheter ablation

Left atrial (LA) remodeling is believed to limit the effectiveness of catheter ablation for atrial fibrillation (AF), as more advanced atrial disease reduces the likelihood of a successful outcome. Traditionally, the degree of atrial remodeling was assessed by measuring the LA diameter. However, recent advancements in contrast-enhanced cardiac magnetic resonance (CE-CMR) allow for a more comprehensive evaluation of the LA, including the identification and quantification of LA fibrosis, precise measurement of LA volume, and assessment of LA shape and sphericity [22]. The present study found a clear association between LA volume, sphericity, and fibrosis: greater LA dilation, more pronounced sphericity, and increased fibrosis. However, these associations were of weak to moderate strength, suggesting that they reflect different underlying processes contributing to atrial remodeling. Notably, LA volume was a stronger predictor of AF recurrence after catheter ablation than both LA sphericity and fibrosis.

Left atrial (LA) passive function occurs in early diastole and represents the “conduit” phase, where blood flows from the pulmonary veins into the left ventricle. In patients with impaired left ventricular relaxation, elevated filling pressures lead to a decline in LA passive function. This may result in LA stretch and pulmonary vein dilation, which increases the risk of atrial fibrillation (AF). Our findings support a mechanistic link between elevated filling pressures (as measured by LAPEF) and late recurrent AF. Given the costs and potential complications of pulmonary vein isolation (PVI), we believe that LAPEF can serve as a physiological marker to help physicians and patients have more personalized discussions regarding the potential benefits of the procedure. A related study shows that over half of patients with LAPEF < 10 % in our sample experienced late recurrent AF, suggesting these individuals are at high risk for recurrence. In contrast, fewer than one in five patients with LAPEF > 40 % experienced recurrence [12].

Recent studies have highlighted left atrial (LA) strain assessment as a more valuable measure of LA function than LA volume in predicting atrial fibrillation (AF) recurrence. Histological analyses have shown that PLAS (plasma left atrial strain) is strongly correlated with the degree of fibrofatty infiltration in the LA wall. The assessment of LA strain includes two main types: LA rapid strain and LA feature tracking strain. LA rapid strain is further subdivided into rapid long-axis strain and AV junction strain, while LA feature tracking strain includes LA reservoir strain, conduit strain, and contractile strain. The study demonstrated that all LA strain parameters—specifically long-axis strain, AV junction strain, and FT reservoir strain—are more susceptible to damage in patients who experience AF recurrence after catheter ablation, compared to those without recurrence. One-way regression analysis indicates that both LA rapid strain and LA feature tracking strain are independent risk factors for AF recurrence after catheter ablation [24]. Contractile strain exhibits the lowest strain values among all LA strain parameters, making it more sensitive to small changes. This could be influenced by the low recurrence rates observed in this cohort. Studies evaluating inter- and intraobserver variability for all three LA strain parameters have shown that contractile strain has better variability compared to reservoir and conduit strain [15].

Several factors may contribute to the worsening of left atrial (LA) function after catheter ablation in patients with recurrent atrial fibrillation (AF). One hypothesis is the natural progression of LA remodeling due to recurrent AF episodes. AF-induced remodeling involves both electrical and mechanical/structural components. While electrical remodeling reverses quickly after AF termination, the mechanical and structural changes take longer to reverse, if they reverse at all. Another potential cause of worsening LA function post-ablation is the formation of ablation-induced scar tissue. It is well established that catheter ablation leads to new scar formation within the LA myocardium. Additionally, studies have shown that a larger extent of LA scarring correlates with worse LA function. In our study, LA function during long-term follow-up was inversely associated with the extent of late gadolinium enhancement (LGE) on cardiac magnetic resonance (CMR). This inverse relationship has also been observed in other studies of patients who underwent AF catheter ablation.

## 5 Conclusions

Short-term left atrial functional impairment can be observed after catheter ablation, while long-term reduction in left atrial volume can be seen. Changes in left atrial volume are likely to lead to the recurrence of atrial fibrillation, while alterations in left atrial function help maintain sinus rhythm. Larger left atrial volume and lower emptying and strain function at baseline assessment by cardiac magnetic resonance are more likely to lead to recurrence of atrial fibrillation after catheter ablation, which may be useful to identify those for whom catheter ablation has reduced success or for whom more aggressive ablation or medications may be useful.

## CRediT authorship contribution statement

**Cuncun Yu:** Data curation, Methodology, Software, Writing-original draft, Writing - review & editing. **zhenjuan Liu:** Supervision, Validation. **shiyu Zhu:** Data curation.

## Declaration of competing interest

The authors declare that they have no known competing financial interests or personal relationships that could have appeared to influence the work reported in this paper.

## References

[1] Van Gelder I.C., Rienstra M., Bunting K.V., Casado-Arroyo R., Caso V., Crijns H. (2024). 2024 ESC Guidelines for the management of atrial fibrillation developed in collaboration with the European Association for Cardio-Thoracic Surgery (EACTS). Eur. Heart J..

[2] Akoum N., Wilber D., Hindricks G., Jais P., Cates J., Marchlinski F. (2015). MRI Assessment of Ablation-Induced Scarring in Atrial Fibrillation: Analysis from the DECAAF Study. J. Cardiovasc. Electrophysiol..

[3] Fukumoto K., Habibi M., Gucuk Ipek E., Khurram I.M., Zimmerman S.L., Zipunnikov V. (2015). Comparison of preexisting and ablation-induced late gadolinium enhancement on left atrial magnetic resonance imaging. Heart Rhythm.

[4] Habibi M., Lima J.A., Khurram I.M., Zimmerman S.L., Zipunnikov V., Fukumoto K. (2015). Association of left atrial function and left atrial enhancement in patients with atrial fibrillation: cardiac magnetic resonance study. Circ. Cardiovasc. Imaging.

[5] Kuppahally S.S., Akoum N., Burgon N.S., Badger T.J., Kholmovski E.G., Vijayakumar S. (2010). Left atrial strain and strain rate in patients with paroxysmal and persistent atrial fibrillation: relationship to left atrial structural remodeling detected by delayed-enhancement MRI. Circ. Cardiovasc. Imaging.

[6] Kowallick J.T., Kutty S., Edelmann F., Chiribiri A., Villa A., Steinmetz M. (2014). Quantification of left atrial strain and strain rate using Cardiovascular Magnetic Resonance myocardial feature tracking: a feasibility study. J. Cardiovasc. Magn. Reson..

[7] Page M.J., McKenzie J.E., Bossuyt P.M., Boutron I., Hoffmann T.C., Mulrow C.D. (2021). The PRISMA 2020 statement: an updated guideline for reporting systematic reviews. BMJ.

[8] Benjamin M.M., Moulki N., Waqar A., Ravipati H., Schoenecker N., Wilber D. (2022). Association of left atrial strain by cardiovascular magnetic resonance with recurrence of atrial fibrillation following catheter ablation. J. Cardiovasc. Magn. Reson..

[9] Hopman L., Mulder M.J., van der Laan A.M., Bhagirath P., Demirkiran A., von Bartheld M.B. (2023). Left atrial strain is associated with arrhythmia recurrence after atrial fibrillation ablation: Cardiac magnetic resonance rapid strain vs. feature tracking strain. Int. J. Cardiol..

[10] Habibi M., Lima J.A.C., Gucuk Ipek E., Spragg D., Ashikaga H., Marine J.E. (2021). Short- and long-term associations of atrial fibrillation catheter ablation with left atrial structure and function: A cardiac magnetic resonance study. J. Cardiovasc. Electrophysiol..

[11] Csécs I., Yamaguchi T., Kheirkhahan M., Czimbalmos C., Fochler F., Kholmovski E.G. (2020). Left atrial functional and structural changes associated with ablation of atrial fibrillation - Cardiac magnetic resonance study. Int. J. Cardiol..

[12] Dodson J.A., Neilan T.G., Shah R.V., Farhad H., Blankstein R., Steigner M. (2014). Left atrial passive emptying function determined by cardiac magnetic resonance predicts atrial fibrillation recurrence after pulmonary vein isolation. Circ. Cardiovasc. Imaging.

[13] Habibi M., Lima J.A.C., Gucuk Ipek E., Zimmerman S.L., Zipunnikov V., Spragg D. (2016). The association of baseline left atrial structure and function measured with cardiac magnetic resonance and pulmonary vein isolation outcome in patients with drug-refractory atrial fibrillation. Heart Rhythm.

[14] Nakamori S., Ngo L.H., Tugal D., Manning W.J., Nezafat R. (2018). Incremental Value of Left Atrial Geometric Remodeling in Predicting Late Atrial Fibrillation Recurrence After Pulmonary Vein Isolation: A Cardiovascular Magnetic Resonance Study. J. Am. Heart Assoc..

[15] Gastl M., Bejinariu A., Behm P., Lindert A., Kelm M., Makimoto H. (2022). Role of CMR-derived atrial deformation analysis in the prediction of atrial fibrillation recurrence rate after pulmonary vein isolation. Eur. J. Radiol..

[16] Ciuffo L., Tao S., Gucuk Ipek E., Zghaib T., Balouch M., Lima J.A.C. (2019). Intra-Atrial Dyssynchrony During Sinus Rhythm Predicts Recurrence After the First Catheter Ablation for Atrial Fibrillation. J. Am. Coll. Cardiol. Img..

[17] Hof I.E., Vonken E.J., Velthuis B.K., Wittkampf F.H., van der Heijden J.F., Neven K.G. (2014). Impact of pulmonary vein antrum isolation on left atrial size and function in patients with atrial fibrillation. Journal of Interventional Cardiac Electrophysiology: an International Journal of Arrhythmias and Pacing..

[18] Chubb H., Karim R., Mukherjee R., Williams S.E., Whitaker J., Harrison J. (2019). A comprehensive multi-index cardiac magnetic resonance-guided assessment of atrial fibrillation substrate prior to ablation: Prediction of long-term outcomes. J. Cardiovasc. Electrophysiol..

[19] Gucuk Ipek E., Marine J.E., Habibi M., Chrispin J., Lima J., Rickard J. (2016). Association of left atrial function with incident atypical atrial flutter after atrial fibrillation ablation. Heart Rhythm.

[20] Jahnke C., Fischer J., Gerds-Li J.H., Gebker R., Manka R., Fleck E. (2011). Serial monitoring of reverse left-atrial remodeling after pulmonary vein isolation in patients with atrial fibrillation: a magnetic resonance imaging study. Int. J. Cardiol..

[21] Poole J.E., Bahnson T.D., Monahan K.H., Johnson G., Rostami H., Silverstein A.P. (2020). Recurrence of Atrial Fibrillation After Catheter Ablation or Antiarrhythmic Drug Therapy in the CABANA Trial. J. Am. Coll. Cardiol..

[22] den Uijl D.W., Cabanelas N., Benito E.M., Figueras R., Alarcón F., Borràs R. (2018). Impact of left atrial volume, sphericity, and fibrosis on the outcome of catheter ablation for atrial fibrillation. J. Cardiovasc. Electrophysiol..

[23] Tsang T.S., Gersh B.J., Appleton C.P., Tajik A.J., Barnes M.E., Bailey K.R. (2002). Left ventricular diastolic dysfunction as a predictor of the first diagnosed nonvalvular atrial fibrillation in 840 elderly men and women. J. Am. Coll. Cardiol..

[24] Benjamin M.M., Moulki N., Waqar A., Ravipati H., Schoenecker N., Wilber D. (2022). Association of left atrial strain by cardiovascular magnetic resonance with recurrence of atrial fibrillation following catheter ablation. J. Cardiovasc. Magn. Reson..

