# Supplementary material for: Progress in the application of cardiac magnetic resonance to predict recurrence of atrial fibrillation after catheter ablation: A systematic review and meta-analysis
Source: Int J Cardiol Heart Vasc. 2025 Jan 17;56:101603. doi: 10.1016/j.ijcha.2025.101603 (PMC11787533; doi:10.1016/j.ijcha.2025.101603)
Supplement: Supplementary Data 1 [file mmc1.docx]

**Supplementary S1.**

**Search strategy:**

(((Spin Echo Imagings) ((((((((((((((((((((((((Catheter Ablation) OR (Ablation, Catheter)) OR (Ablation, Transvenous Electric)) OR (Electric Ablation, Transvenous)) OR (Transvenous Electric Ablation)) OR (Ablation, Transvenous Electrical)) OR (Electrical Ablation, Transvenous)) OR (Transvenous Electrical Ablation)) OR (Catheter Ablation, Electric)) OR (Electric Catheter Ablation)) OR (Ablation, Electric Catheter)) OR (Catheter Ablation, Electrical)) OR (Ablation, Electrical Catheter)) OR (Electrical Catheter Ablation)) OR (Catheter Ablation, Percutaneous)) OR (Percutaneous Catheter Ablation)) OR (Ablation, Percutaneous Catheter)) OR (Catheter Ablation, Radiofrequency)) OR (Radiofrequency Catheter Ablation)) OR (Ablation, Radiofrequency Catheter)) OR (Catheter Ablation, Transvenous)) OR (Transvenous Catheter Ablation)) OR (Ablation, Transvenous Catheter)) OR ("Catheter Ablation"[Mesh]))) AND ((((((((((((((((((((((((((("Atrial Fibrillation"[Mesh]) OR (Atrial Fibrillation)) OR (Atrial Fibrillations)) OR (Fibrillation, Atrial)) OR (Fibrillations, Atrial)) OR (Auricular Fibrillation)) OR (Auricular Fibrillations)) OR (Fibrillation, Auricular)) OR (Fibrillations, Auricular)) OR (Persistent Atrial Fibrillation)) OR (Atrial Fibrillation, Persistent)) OR (Atrial Fibrillations, Persistent)) OR (Fibrillation, Persistent Atrial)) OR (Fibrillations, Persistent Atrial)) OR (Persistent Atrial Fibrillations)) OR (Familial Atrial Fibrillation)) OR (Atrial Fibrillation, Familial)) OR (Atrial Fibrillations, Familial)) OR (Familial Atrial Fibrillations)) OR (Fibrillation, Familial Atrial)) OR (Fibrillations, Familial Atrial)) OR (Paroxysmal Atrial Fibrillation)) OR (Atrial Fibrillation, Paroxysmal)) OR (Atrial Fibrillations, Paroxysmal)) OR (Fibrillation, Paroxysmal Atrial)) OR (Fibrillations, Paroxysmal Atrial)) OR (Paroxysmal Atrial Fibrillations))) AND ((((((((((((((((((((((((((((((((((((((((((("Magnetic Resonance Imaging"[Mesh]) OR (magnetic resonance imaging)) OR (Imaging, Magnetic Resonance)) OR (NMR Imaging)) OR (Imaging, NMR)) OR (Zeugmatography)) OR (Tomography, MR)) OR (Steady-State Free Precession MRI)) OR (Steady State Free Precession MRI)) OR (NMR Tomography)) OR (Tomography, NMR)) OR (MR Tomography)) OR (Tomography, Proton Spin)) OR (Proton Spin Tomography)) OR (Magnetization Transfer Contrast Imaging)) OR (fMRI)) OR (Magnetic Resonance Imaging, Functional)) OR (MRI, Functional)) OR (Functional MRI)) OR (Functional MRIs)) OR (MRIs, Functional)) OR (Functional Magnetic Resonance Imaging)) OR (MRI Scans)) OR (MRI Scan)) OR (Scan, MRI)) OR (Scans, MRI)) OR (imaging, Chemical Shift)) OR (maging, Chemical Shift)) OR (Chemical Shift Imagings)) OR (Imagings, Chemical Shift)) OR (Shift Imaging, Chemical)) OR (Shift Imagings, Chemical)) OR (Chemical Shift Imaging)) OR (Spin Echo Imaging)) OR (Echo Imaging, Spin)) OR (Echo Imagings, Spin)) OR (Imaging, Spin Echo)) OR (Imagings, Spin Echo)) OR (Spin Echo Imagings)) OR (Magnetic Resonance Image)) OR (Image, Magnetic Resonance)) OR (Magnetic Resonance Images)) OR (Resonance Image, Magnetic))

Forest plot of the number of paroxysmal AF in AF recurrence and No AF recurrence
